# Prediction of glioma-subtypes: comparison of performance on a DL classifier using bounding box areas versus annotated tumors

**DOI:** 10.1186/s42490-022-00061-3

**Published:** 2022-05-19

**Authors:** Muhaddisa Barat Ali, Irene Yu-Hua Gu, Alice Lidemar, Mitchel S. Berger, Georg Widhalm, Asgeir Store Jakola

**Affiliations:** 1 Department of Electrical Engineering, Chalmers University of Technology, Gothenburg, Sweden; 2 Department of Clinical Neuroscience, University of Gothenburg, Gothenburg, Sweden; 3 Department of Neurological Surgery,, University of California San Francisco, San Francisco, USA; 4 Department of Neurosurgery, Medical University of Vienna, Vienna, Austria; 5 Department of Neurosurgery, Sahlgrenska University Hospital, Gothenberg, Sweden

**Keywords:** 1p/19q codeletion, IDH genotype, Brain tumor, Ellipse bounding box, Deep learning

## Abstract

**Background:**

For brain tumors, identifying the molecular subtypes from magnetic resonance imaging (MRI) is desirable, but remains a challenging task. Recent machine learning and deep learning (DL) approaches may help the classification/prediction of tumor subtypes through MRIs. However, most of these methods require annotated data with ground truth (GT) tumor areas manually drawn by medical experts. The manual annotation is a time consuming process with high demand on medical personnel. As an alternative automatic segmentation is often used. However, it does not guarantee the quality and could lead to improper or failed segmented boundaries due to differences in MRI acquisition parameters across imaging centers, as segmentation is an ill-defined problem. Analogous to visual object tracking and classification, this paper shifts the paradigm by training a classifier using tumor bounding box areas in MR images. The aim of our study is to see whether it is possible to replace GT tumor areas by tumor bounding box areas (e.g. ellipse shaped boxes) for classification without a significant drop in performance.

**Method:**

In patients with diffuse gliomas, training a deep learning classifier for subtype prediction by employing tumor regions of interest (ROIs) using ellipse bounding box versus manual annotated data. Experiments were conducted on two datasets (US and TCGA) consisting of multi-modality MRI scans where the US dataset contained patients with diffuse low-grade gliomas (dLGG) exclusively.

**Results:**

Prediction rates were obtained on 2 test datasets: 69.86% for 1p/19q codeletion status on US dataset and 79.50% for IDH mutation/wild-type on TCGA dataset. Comparisons with that of using annotated GT tumor data for training showed an average of 3.0% degradation (2.92% for 1p/19q codeletion status and 3.23% for IDH genotype).

**Conclusion:**

Using tumor ROIs, i.e., ellipse bounding box tumor areas to replace annotated GT tumor areas for training a deep learning scheme, cause only a modest decline in performance in terms of subtype prediction. With more data that can be made available, this may be a reasonable trade-off where decline in performance may be counteracted with more data.

## Introduction

The most common type of brain tumor is called diffuse glioma and is the reason of 80% of malignant brain tumors [1]. Depending on the aggressiveness of the tumor, World Health Organization (WHO) has categorized them into grades 2-4 where higher grade means more malignant tumors, and classified as either astrocytomas and oligodendrogliomas [2]. Traditionally, the grade 2 gliomas are referred to as low-grade gliomas (LGG) and grade 3-4 as high-grade gliomas (HGG). Additionally, recent findings on molecular biomarkers have revised WHO grading to its further subtypes. According to this, isocitrate dehydrogenase (IDH) mutation and 1p/19q codeletion are the hallmarks of the dLGG subtypes which beyond classification also provides important information concerning prognosis and response to therapy [3]. IDH mutations are detected in 70-80% of dLGG [4]. The survival rate for dLGG IDH mutated patients are higher than IDH wild-type patients and plays an important role in prognosis and clinical decisions. This observation has also caused dLGG IDH wild-type with molecular features of glioblastoma to be classified as gliobalstomas [5]. Also, in IDH mutated astrocytomas the prognostic importance of extensive cytoreductive surgery is highly convincing [6–9]. Codeletion of 1p/19q is a characteristic of oligodendrogliomas and is a favourable prognostic molecular marker. Since oligodendrogliomas are more sensitive to oncological treatment [8, 9], the role of extensive resection have been discussed and surgical management could be directly affected by knowing dLGG subtype. Therefore, precisely knowing the molecular marker prior to surgery would be of practical value. Recently non-invasive classification methods have shown promising results in prediction of glioma-subtypes based upon pre-operative imaging [10–13]. Non-invasive methods are opening up to discuss tailored therapies that would assist the surgeons and patients in the shared decision making process [14]. However, many challenges remain before bringing these tools into clinical practice.

Accurate tumor boundaries are important, since pixels within tumor boundaries are labeled as tumor for the supervised training of glioma. Using incorrectly labeled pixels for supervised training could lead to reduced test performance of classifier for distinguishing tumors. This pre-processing step helps more accurate supervised training of tumor tissues. Drawing tumor boundaries manually by medical experts is a tedious task often requiring clinicians with anatomical and physiological expertise. Apart from being time consuming task, it makes this procedure prone to intra and inter observer variability [15, 16]. Automatic segmentation is an alternate way to manual annotation. Studies have been conducted for automatic and semi-automatic segmentation of tumors to overcome the time and radiologist constraints e.g. support vector machine [17], decision tree [18], conditional random forest [19], mean shift [20], graph cut algorithm [21], level set method [22] and many more. Recently, DL has gained much attention for its high performance in segmentation of medical images [23, 24]. The most frequently used model for characterizing visual objects and learning dense characteristics of images is Convolutional Neural Network (CNN) [25]. Relevant works that include segmentation are, among others, U-net [26], patch-based CNN [27] or patch-based multi-scale CNN [28]. However, these methods do not guarantee the quality and could lead to improper or failed segmented boundaries making the segmentation process an ill-defined problem. These approaches are often dependent on the quality and representation of the features and sometimes require physician intervention to identify the most important features, if automatic segmentation fails [25]. Moreover, the devices and protocols used for acquisition of MRIs can vary dramatically on brain scans causing intensity biases and other variations of brain scans in a dataset. These issues may lead to ambiguous ROIs (regions of interest) in segmentation that subsequently affect the diagnosis or classification. Most deep learning segmentation methods are based on supervised learning which requires annotated GT (ground truth) tumor regions for training. Many medical imaging datasets lack the GT tumor annotations that limits the use of those datasets. It is worth noting that using bounding boxes for object tracking [29] and classification have been successfully applied on visual images to bypass the ambiguity issues in automatic segmentation. However, this idea is rarely applied in MRI-based diagnosis.

For glioma classification, DL offers an automatic way to learn features. In the past few years, several DL methods have been successfully introduced for such applications. Chang et al. [30] introduced a method that uses residual CNNs for the prediction of IDH mutation using four modalities of MRI data. Li et al. [31] trained a 6 layer CNN for tumor segmentation on GT tumor data. Then features from the last fully connected layer were size normalized by Fisher vector coding followed by a SVM classifier for IDH mutation prediction. Liang et al. [32] suggested to use more advanced DenseNets using 3D MRI scans for IDH mutation prediction and obtained good performance for glioma grading. Yogananda et al. [13] proposed an approach on training from scratch 3D-Dense-UNets for performing classification and segmentation simultaneously for IDH mutation status and proved that network trained on FLAIR-MRIs gives the same performance as when trained on multi-contrast MRIs (T2, FLAIR and T1ce) on TCGA dataset with 214 patients. Then, in [12] they used the same trained network in transfer learning for 1p/19q codeletion prediction with 368 patients with T2-MRIs.

Our work is mainly motivated for the prediction of diffuse glioma-subtypes by shifting the paradigm in supervised training by using tumor ROIs specified by bounding box areas e.g., ellipse shaped around the tumors instead of accurate tumor boundaries. Although, manual GT annotation has been the best way to allocate ROIs, it is a time consuming process and needs medical expertise. Likewise, automatic segmentation comes with its own challenges because it is an ill-defined problem and doesn’t always guarantee accurate tumor boundaries. Inspired by computer vision community’s successful research on visual object tracking and classification using bounding boxes, this paper attempts to shift the study through an alternate paradigm for MRI-tumor subtypes prediction where supervised training in DL scheme utilizes the bounding box areas on MRI medical data. To the best of our knowledge, it is the first time that such a strategy has been successfully adopted for diffuse glioma-subtype prediction and comparing the performance to those trained on GT tumor areas. In this work, we used tight ellipse bounding boxes for locating brain tumor areas, in such a way that surrounding tissue does not cause much deterioration of the features in identifying the subtypes of diffuse gliomas. We show that a glioma-subtype classifier trained by using tumor bounding box areas may achieve comparable performance, with a slight performance degradation of about 3.0% averaged on 2 dataset results.

## Overview of a DL classification scheme: 2D multi-stream CNN classifier

**Fig. 1:**
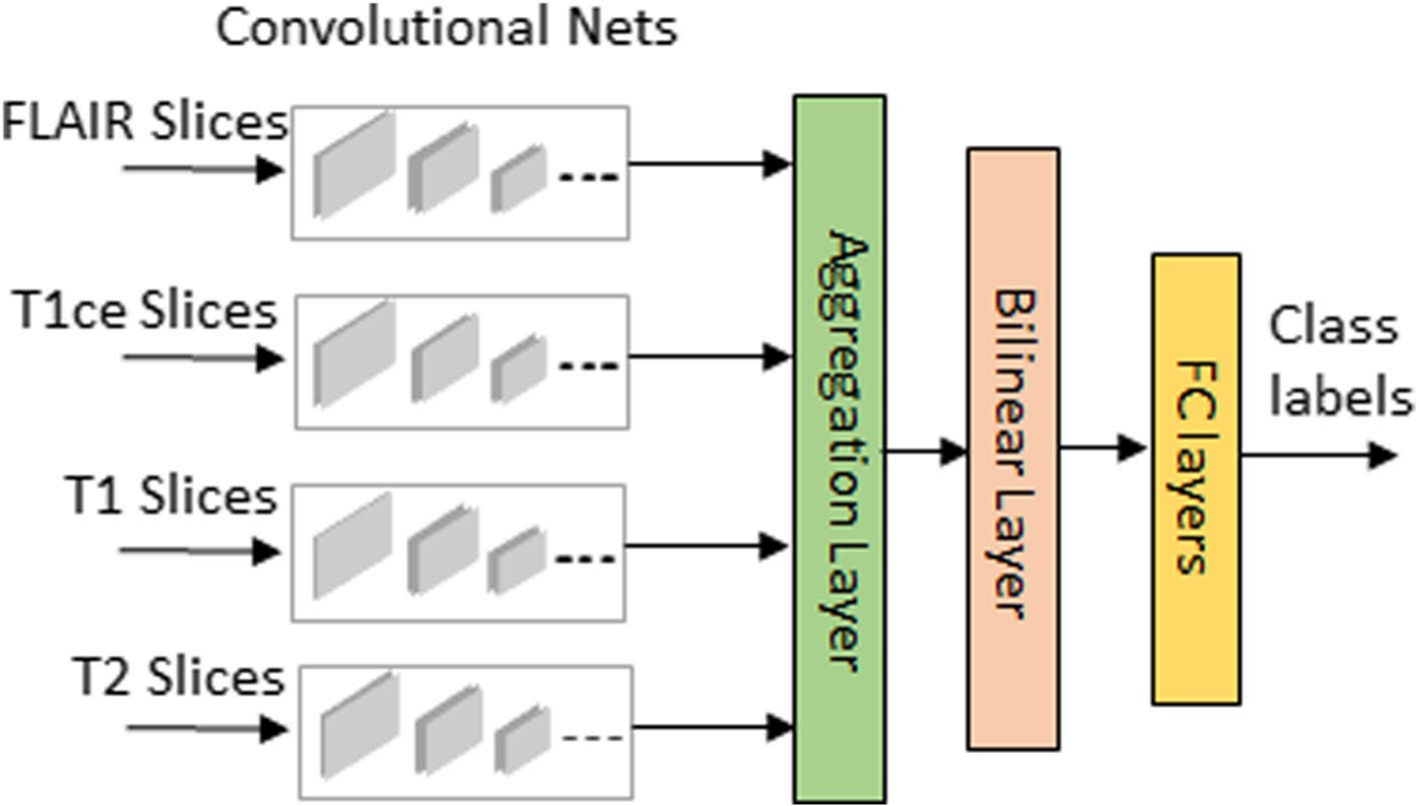
Multi-stream 2D convolutional neural network for glioma-subtype classification from [46]

We adopted the classifier from a previous work [46] as a DL prediction scheme for the feature learning and classification of glioma-subtypes. Considering the moderate and small sizes of training datasets, we choose a 2D MRI slice-based classifier as: (a) due to the curse of dimensionality, one has to significantly increase the size of MRI training dataset to avoid the over-fitting, if high dimensional 3D volume data is used as the input; (b) using slice based approach could significantly reduce the computations by only processing a few slices containing the tumor. For the sake of convenience to the readers, a brief overview of the classifier is given in Fig. 1. The deep network uses number of streams based on the MRI modalities used. Each stream consists of 6 convolutional layers with filter size 3 ×3 in each layer. Let the feature maps with their modality specific characteristics from all streams be denoted as *F*_1_,*F*_2_,*F*_3_ and *F*_4_ respectively. These features are extracted from the last convolutional layers followed by the feature information fusion layers, where the features are fused together as *F*=*F*_1_⊙*F*_2_⊙*F*_3_⊙*F*_4_ at aggregation layer and are compactly represented at bilinear layer as *y*=*F*^*T*^*F*. The final refined feature map is followed by 2 fully connected layers with random initialization and dropout regularization that ends at a final layer for glioma-subtype class prediction.

## Proposed method

In this section, we describe the proposed approach where tumor ROIs are employed as the inputs for training the DL scheme. First, the approach for tumor subtype prediction and performance comparison are described. Then the selection of ellipse bounding boxes as tumor ROIs is described.

### Glioma-subtype prediction based on the DL scheme trained by tumor ROIs

**Fig. 2:**
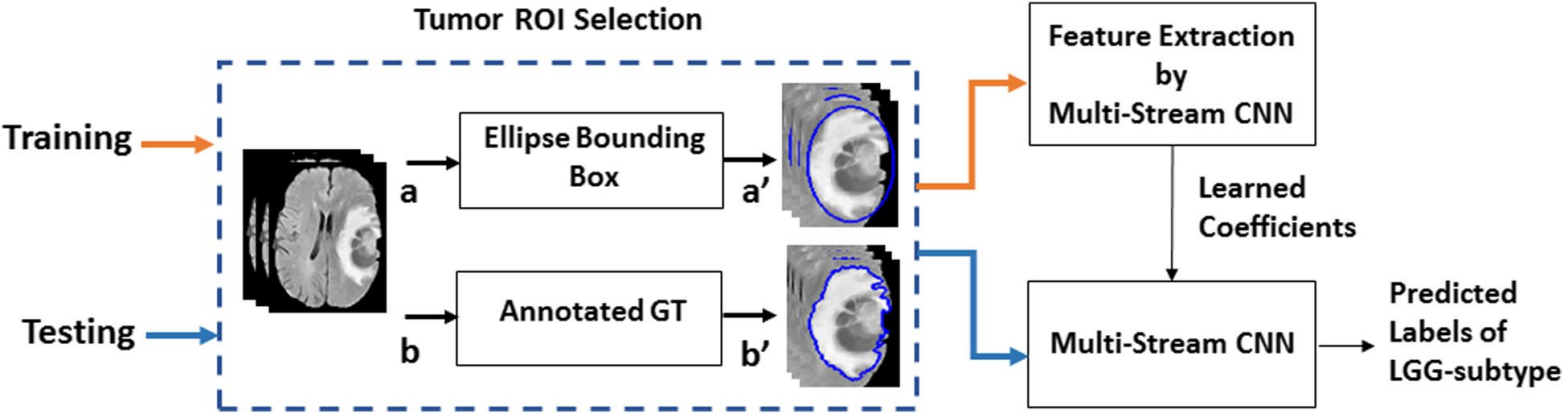
The pipeline of the method based on proposed strategy. Blue dash box: Tumor areas separated by ellipse bounding box and manually drawn GT boundary. **Orange arrow:** Training phase. **Blue arrow:** Testing phase

The proposed strategy introduces ellipse shaped bounding boxes as ROIs to occupy all the tumor areas. Figure 2 shows the block diagram of the pipeline for glioma-subtype prediction based on two datasets: TCGA (public dataset) and US (clinical dataset). Two modalities (FLAIR and T1ce) from US dataset are used for case study-A and four modalities (T1, T2, FLAIR and T1 contrast enhanced (T1ce)) are used from TCGA dataset for case study-B. In order to find how well the proposed strategy performs compared to that of using GT annotated data, a comparison of the performance of the classifier was examined against each training data type. Firstly, the classifier was trained and tested on ellipse tumor bounding box data. Secondly, the same experiment was repeated by training on the manually annotated GT tumor data.

From Fig. 2, input 2D multi-modality MRIs are fed to tumor ROI selection block, as shown in blue dotted box. When this block receives input 2D MR images from point **a**, it processes them to output point **a’** by introducing a tight ellipse bounding box around the tumor area. Then, the multi-stream 2D Convolutional neural network is trained on the selected ROIs to learn features from each of the corresponding multi-modality MRIs. After the model is trained, during testing phase, the prediction is obtained from the test data with ellipse tumor bounding box areas obtained at point **a’**. To check the classification performance with that trained by GT annotated data, MRIs are given at point **b** for GT ROIs selection and are processed further at point **b’** in the blue dashed block. Following this, the network is trained and tested accordingly. Finally, the test accuracy on both the data types are compared. This procedure is repeated separately for each of the datasets.

### Tumor ROI selection: ellipse bounding box

**Fig. 3:**
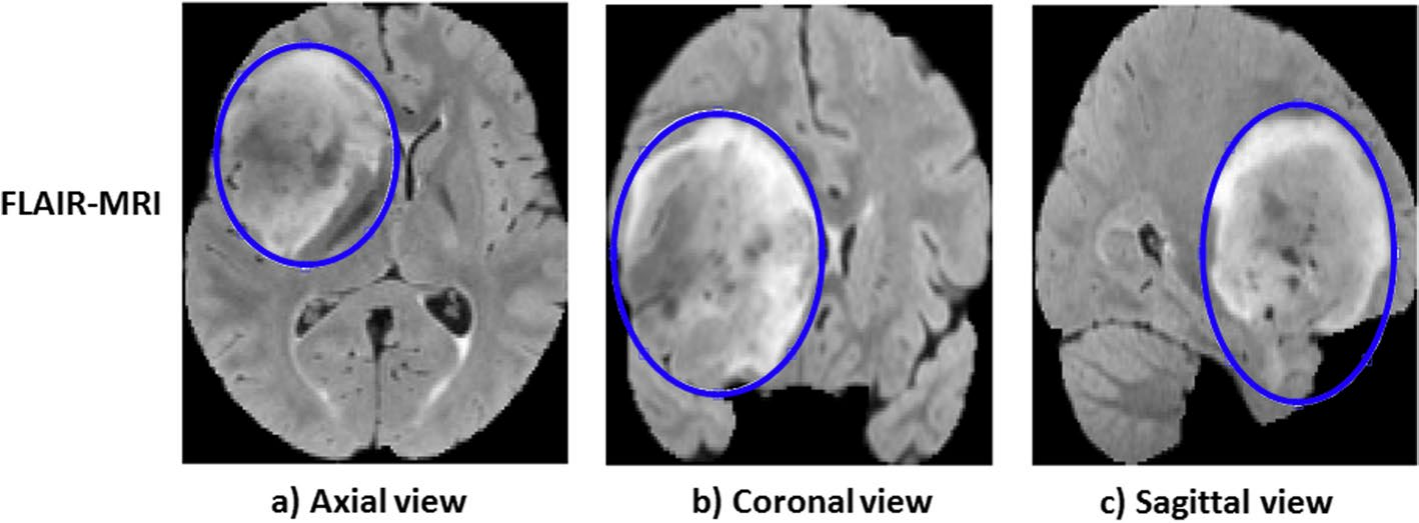
Illustration of selection of ROIs with tight ellipse bounding box for a FLAIR-MRI from US dataset for all three directional views. The blue line defines the tumor area contour

In this part, we shall give further details of the blue dashed box from Fig. 2. DL is computationally expensive and brain MRIs are complex that consist of many anatomical details. Typically, a full 2D slice image isn’t useful to detect subtype of gliomas on molecular level. The tumor areas can be better focused for a faster and more accurate model training. As brain tumors show great variations in shape, size and intensity, a tight elliptical shaped bounding box is introduced surrounding the tumor. In this work, tight elliptical bounding boxes are obtained manually. As mentioned in [34–36], we believe that this strategy helps to capture certain amount of information not only in tumor region but also information from the surrounding tissue that may not cause a major problem in recognition of glioma-subtypes. Tumor area selection using ellipse bounding box is shown in Fig. 3 for 3 directional views of a FLAIR-MRI. As FLAIR-MRIs present visually better tumor contrast with its surrounding tissues, a tight ellipse bounding box is drawn manually with the help of 8 points whose positions are adjusted in accordance with the shape of the tumor. The binary tumor mask generated from this procedure is then applied to the other modalities of the patient to generate ellipse shaped tumor data for all modalities.

**Fig. 4:**
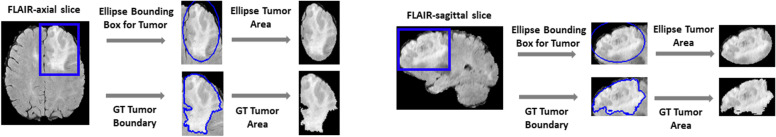
An example of TCGA dataset from IDH mutation class. Separation of ROIs is shown in both ways (using ellipse bounding box and GT) on FLAIR modality. **Left:** Axial view. **Right:** Sagittal view

From Fig. 2, the other way to separate the tumor region is by simply masking out the tumor area if the GT annotation is available for the dataset. This generates GT data with manually drawn tumor boundary discarding the surrounding non-tumor tissues. In Fig. 4, examples on ellipse boxed tumor area and GT annotated area are shown for FLAIR-MRI modality.

## Results and comparisons

**Setup:** Experiments were implemented using Keras library [33] with Tensor Flow backend on a workstation with Intel-i7 3.40GHz CPU, 48G RAM and an NVIDIA Titan Xp 12GB GPU. By tuning the network carefully through experiments, different parameters were selected. Learning rate was set to 1.0*e*^−4^. Optimizer used was *Adagrad*. Batch size was set to 16. We used *L*2-norm regularization with the value of parameter selected as 1.0*e*^−4^ for convolutional layers of each stream. The categorical cross-entropy was used as a loss function for evaluating the final performance. Here, we adapted early stopping strategy when the best validation performance was achieved. The random dropout rate was set to 0.5 for two fully connected layers for TCGA dataset and 0.6 for US dataset. Simple data augmentations such as horizontal flipping and random rotation (maximum at 10^∘^) were used by Keras function *ImageData Generator* in real time to minimize the memory usage during training.

**Datasets:** Two datasets were used in the experiments for glioma-subtype prediction as shown in Table 1. One is a clinical dataset from Department of Neurosurgery, University of San Francisco, California (UCSF), referred to the US dataset in this paper. The other is TCGA dataset from TCGA-GBM (n = 101) [37] and TCGA-LGG (n = 66) [38] with IDH genotype labels. The MRI-modalities and the number of patients used for each of the datasets are given in Table 1(a). Unlike TCGA dataset, US dataset consists of only dLGG (Who grade 2) with the typical appearance of non-enhancing hyper-intensive ROIs in FLAIR images and without significant contrast enhancement. The ground truth annotation or tumor mask for TCGA dataset is publicly available. For US dataset, tumor boundaries were drawn manually through the help of 3D slicer tool (v4.10.2) [39] and all annotation was controlled by the senior medical doctor (ASJ), having extensive experience in LGG research and segmentation. The datasets consist of 3D brain scans where 2D image slices from all three views (axial, sagittal and coronal) were extracted for our experiments. Each dataset was split patient wise into 3 sets: training (60%), validation (20%) and testing (20%) such that no images of a patient from one set is used in another. For each run, patients were selected randomly for each of the sets and the results of multiple runs were averaged for the final performance evaluation.

**Table 1:** Summary of Two Datasets (a) Number of 3D scans in each datasets. (b) Description of data for two case studies

**(a)**				
**Dataset**	**T1ce**	**FLAIR**	**T1**	**T2**
US	75	75	-	-
TCGA	167	167	167	167
**(b)**				
**Case Study**	**Glioma Subtype**	**#3D/2D**^**∗**^**(Training)**	**#3D/2D**^**∗**^**(Validation)**	**#3D/2D**^**∗**^**(Testing)**
A	1p/19q cod	25/450	8/144	9/162
	1p/19q non-cod	20/360	6/108	7/126
B	IDH-mut	33/594	11/198	11/198
	IDH-wt	68/612	22/198	22/198

*Excluded with augmented slice images

The details of two case studies are shown in Table 1(b). For case study-A, we used US dataset that has two modalities T1ce-MRI and FLAIR-MRI for prediction of LGG with 1p/19q codeletion and non-codeletion. Here, 42 patients are 1p/19q codeleted and 33 patients are non-codeleted. Observing that the tumor size varies from small to medium in different subjects, 6 slices for each of the views (axial, coronal, sagittal) have been extracted from a 3D scan. Keeping the slice with the biggest tumor area as centre slice, other slices were extracted from both sides. For case study-B, we used TCGA dataset with four modalities (T1ce, FLAIR, T1 and T2) for classifying IDH genotype. For this case study, one can see that 55 patients are labeled as IDH-mutated and 112 patients as IDH-wild type from Table 1(b). Unlike Case study-A, this dataset has large class imbalance for IDH genotype. Therefore, 3 times more slices have been extracted for patients with IDH mutation i.e; 3 for each view for IDH wild-type and 6 for each view for IDH-mutation.

**Criteria:** To evaluate the performance of diffuse glioma-subtype prediction on both case studies, we used accuracy, precision, specificity, sensitivity/recall and F1-score as the evaluation criteria on the test results averaged over 5 runs. The metrics computed were based on the following four kinds of samples:

*True positive (TP):* 1p/19q codeleted/IDH mutated glioma was correctly classified as 1p/19q codeleted/ IDH mutated.

*False positive (FP):* 1p/19q non-codeleted/IDH wild-type glioma was incorrectly classified as 1p/19q codeleted/ IDH mutated.

*True negative (TN):* 1p/19q non-codeleted/IDH wild-typ glioma was correctly classified as 1p/19q non-codeleted/ IDH wild-type.

*False negative (FN):* 1p/19q codeleted/ IDH mutated glioma was incorrectly classified as 1p/19q non-codeleted/ IDH wild-type.

defined as accuracy, specificity and sensitivity.

$$\text{Accuracy} = \frac{\text{TP}+ \text{TN}}{\text{TP} + \text{FP} + \text{TN} + \text{FN}},\quad \text{Precision} = \frac{\text{TP}}{\text{TP} + \text{FP}}$$

$$\text{Specificity} =\frac{\text{TN}}{\text{FP} + \text{TN}}, \quad \text{Sensitivity/Recall} = \frac{\text{TP}}{\text{TP} + \text{FN}}$$

$$\text{F1-score} = 2 \times \frac{(\text{Recall} \times \text{Precision})}{\text{Recall} + \text{Precision}}$$

**Pre-processing:** This step has an impact on the performance. The clinical 3D volume data in US dataset was unregistered. So, the anatomical images from FLAIR and T1ce scans were registered to 1mm MNI space template. In addition to this, the bias field correction and skull-stripping steps were performed using FSL [40] and ANTs [41] tools. The TCGA data needs no pre-processing and is readily available as skull-striped and co-registered with IDH genotype labels. To save computation, slices were rescaled to a 128×128 size and then normalized to range [0,1].

### Results on test sets

First we evaluated the procedure on both case studies/datasets using the classification scheme with the ellipse bounding box tumor data.

**Fig. 5:**
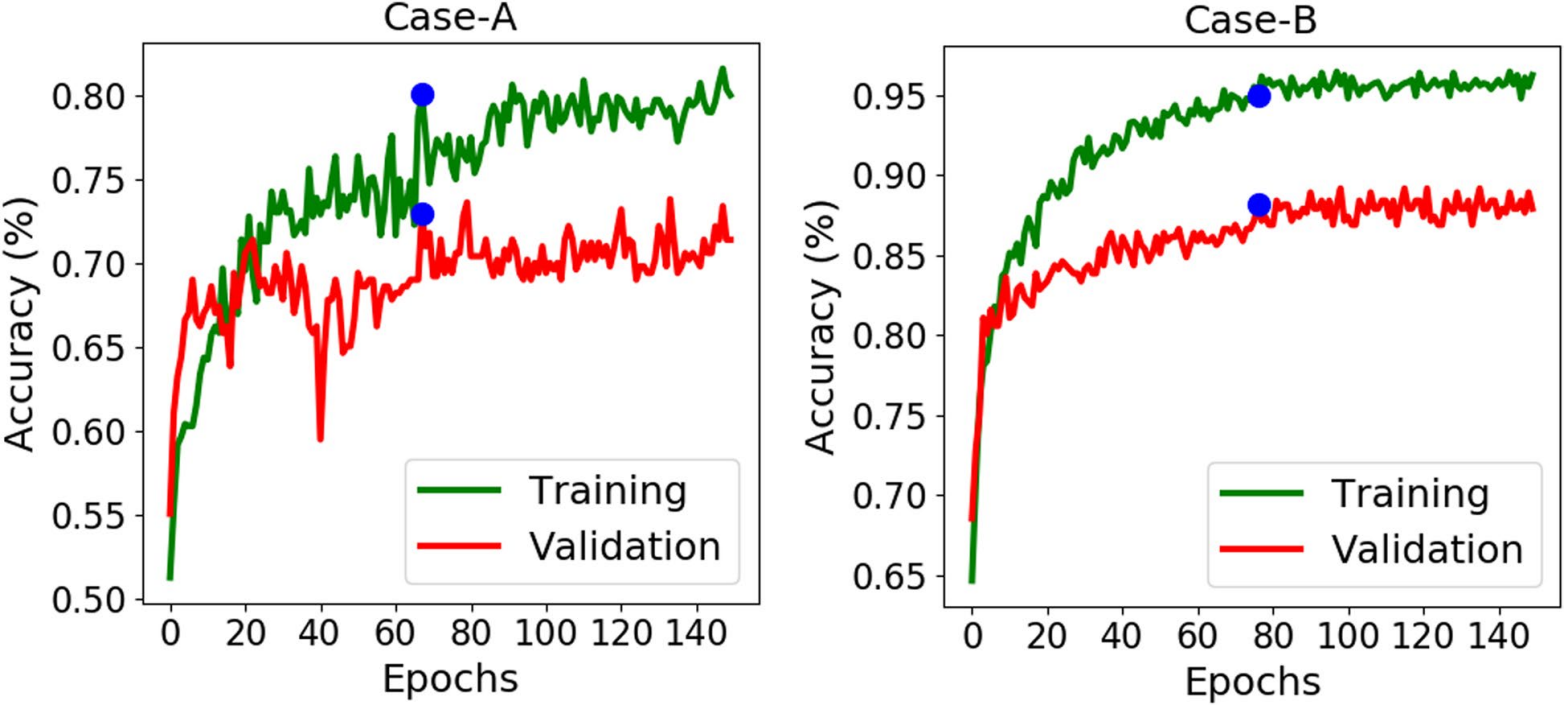
Training (green) and validation (red) curves on ellipse bounding box tumor data as a function of epochs for both case studies. Early stopping strategy was used, where blue dot points to the epoch whose parameters were used for test set. **Left:** For US dataset, the validation curve converged at epoch = 67. **Right:** For TCGA dataset, the validation curve converged at epoch = 76, after which the validation losses didn’t improve

**Case-A:** In this case, US dataset containing only dLGG, was studied. As the data size is small, to help the network learn the features, we used a higher rate of dropout (60%) in the fully connected layers as a regularization effect. Hence, the training and validation curves show up some variations. Figure 5 shows the training and validation curves as a function of epochs for 5^*th*^ run from Table 2(a). Early stopping was applied as one can see from the curve that the validation accuracy did not improve after epoch = 67, hence the coefficients of DL scheme were frozen from this epoch. The testing accuracy obtained was **72.57%** at this epoch. Table 2(a) shows the results on the test dataset. The average test accuracy for 1p/19q prediction is **69.86%**. The average sensitivity 74.20% is higher than the specificity 64.60%, because patients with 1p/19q codeletion are more frequent in this dataset. This resulted an average F1-score of 73.51%.

**Table 2:** Comparison of the average test results for diffuse glioma-subtypes using ellipse bounding box tumor data for 5 runs. The highest values obtained in each run are displayed in bold text. (a) Case-A for US dataset (1p/19q prediction). (b) Case-B for TCGA dataset (IDH genotype)

Run	Dataset	Accuracy (%)	Precision (%)	Sensitivity(%)	Specificity(%)	F1-Score(%)
(a) Case-A: Prediction Result on Ellipse Bounding Tumor Areas						
1		65.97	70.00	69.14	61.90	69.57
2	US	71.53	74.10	75.93	65.87	75.00
3	(1p/19q Codel/	68.06	72.73	69.14	66.67	70.90
4	Non-Codel)	71.18	74.25	76.54	65.87	75.38
5		**72.57**	**73.45**	**80.25**	**62.70**	**76.70**
	Average(∣*σ*∣)	69.86 (2.46)	72.91(1.55)	74.20(4.39)	64.60 (1.92)	73.51(2.76)
(b) Case-B: Prediction Result on Ellipse Bounding Tumor Areas						
1		79.55	85.03	71.71	87.37	77.80
2	TCGA	76.01	78.45	71.72	80.30	74.93
3	(IDH mut/	80.30	86.23	72.73	87.88	78.91
4	wild-type)	**82.58**	**88.69**	**75.25**	**89.90**	**81.42**
5		79.04	85.80	70.20	87.88	77.22
	Average(∣*σ*∣)	79.50(2.12)	84.84(3.42)	72.32(1.67)	86.65(3.28)	78.06 (2.13)

**Case-B:** As observed in Fig. 5 for 4^*th*^ run from Table 2(b), the test accuracy obtained was **82.58%** at epoch = 76. Observing the average prediction result of this case study from Table 2(b), the average sensitivity (72.32%) lower than the average specificity (86.65%) because of the high class imbalance in this dataset between IDH mutated and IDH wild-type class. The average accuracy is **79.50%** and average F1-score as 78.06%. Here, due to large class imbalance F1-score can be considered a better metric for the evaluation.

### Comparison of prediction results with the annotated GT data

**Fig. 6:**
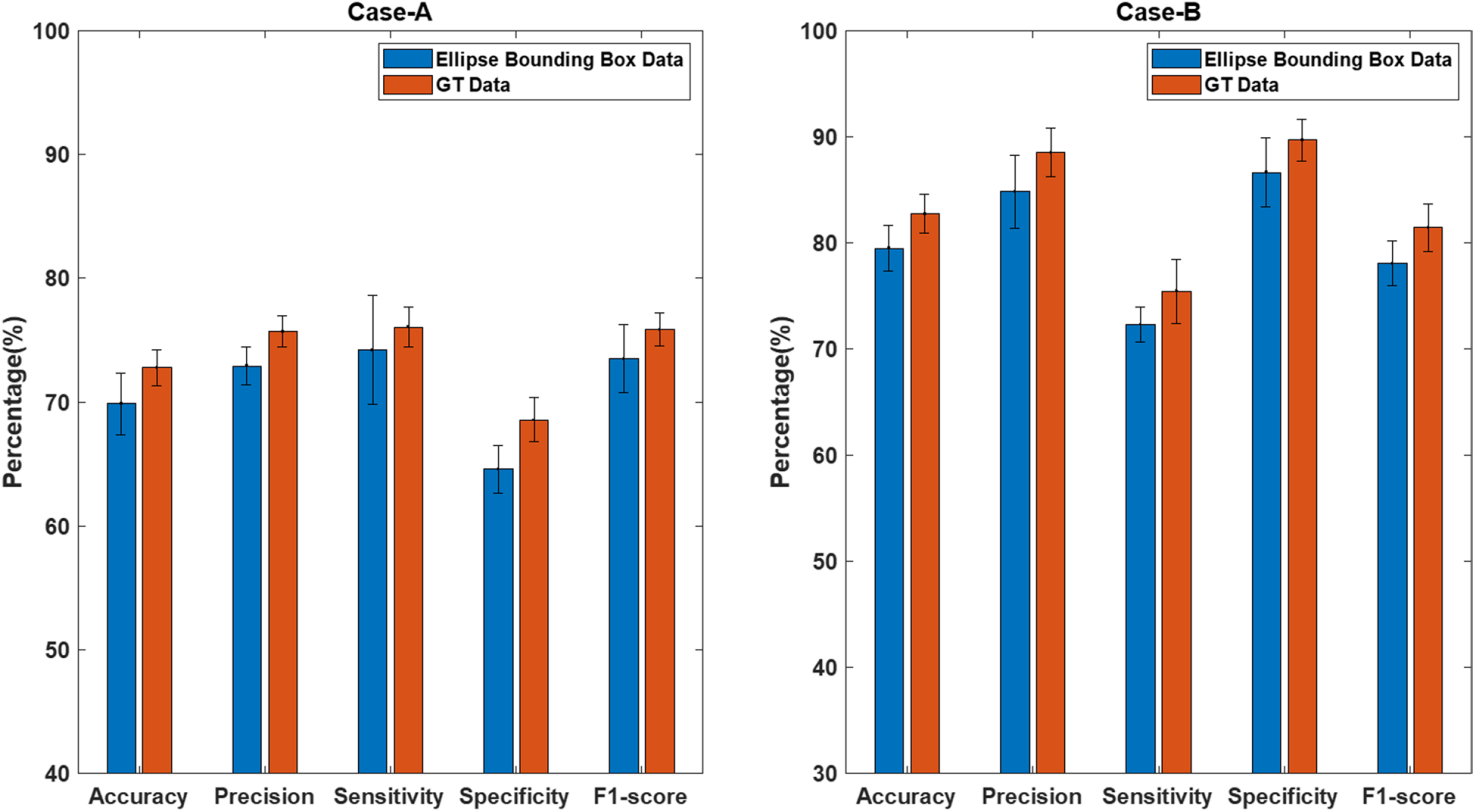
Summary of the evaluation metrics and comparison of prediction on ellipse bounding box data and GT data using multi-stream CNN scheme. **Left: Case-A:** Comparison on US dataset. **Right: Case-B:** Comparison on TCGA dataset

We then compare the prediction performance through otherwise identical DL pipeline, but using annotated GT test sets where the DL scheme was trained by annotated GT training data. The summary of the average performance metrics (all averaged over 5 runs through same sequence of data re-partition for each run and re-training the DL scheme) is shown in Fig. 6. Observing the results for the difference in performance in Table 3, one can see that the average test accuracy with ellipse bounding box has resulted in slightly degraded performance on the test datasets, by 2.92% in US dataset (with difference of 1.85% in sensitivity and 3.97% in specificity) and by 3.23% in TCGA dataset (with difference of 3.05% in sensitivity and 3.13% in specificity).

**Table 3:** Performance difference on average prediction results (over 5 runs) by using GT tumor data and ellipse tumor bounding box data for training, where the standard deviation is included in (·) after each performance value

Case Study	Tumor Area	Av. Acc.(∣*σ*∣)	Av. Sen.(∣*σ*∣)	Av. Spec.(∣*σ*∣)
A	Ellipse	69.86(2.46)	74.20(4.39)	64.60(1.92)
	GT	72.78(1.45)	76.05(1.63)	68.57(1.78)
Difference		2.92(1.45)	1.85(1.78)	3.97(1.63)
B	Ellipse	79.50(2.12)	86.65(3.28)	72.32(1.67)
	GT	82.73(1.82)	89.70(2.00)	75.45(3.04)
Difference		3.23(0.3)	3.05(1.28)	3.13(1.37)

**Fig. 7:**
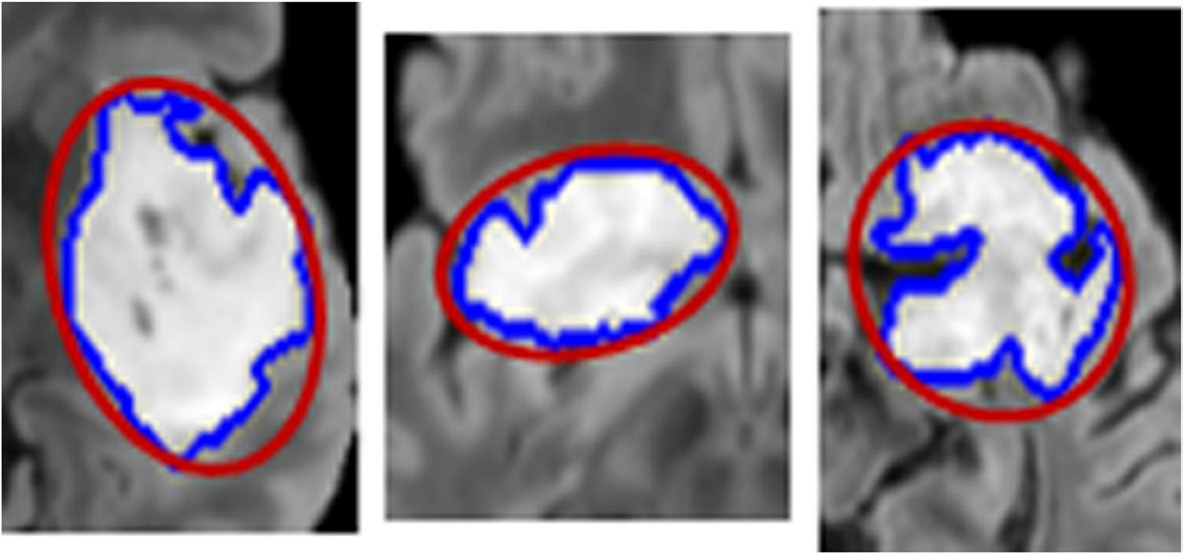
Example images of zoom in brain tumor MRIs. Blue curves are the GT tumor boundaries manually drawn by medical experts, and red curves are the ellipse bounding boxes surrounding the tumors

To further examine the difference between the ellipse bounding box areas and the GT tumor boundaries marked by medical experts (see Fig. 7), the average tumor dice scores were computed on the training sets of the two datasets. The dice score is defined as $D = \frac {2 |X \bigcap Y|}{|X|+|Y| }$, where *X* is the tumor image with pixels within the ellipse area, and *Y* is the GT tumor image with pixels within the GT tumor boundaries. Table 4 shows the average of dice scores on the training sets which indicates that some non-tumor pixels were included in ellipse bounding boxes. This is expected as tumor shape is non elliptical (see Fig. 7, where both GT tumor areas and the ellipse bounding boxes are marked on images). This is rather encouraging, as it indicates that replacing medical experts’ marked GT tumor areas by ellipse bounding box areas in the training has resulted in small performance reduction in the classification (see Table 3) on test sets. Further, it is worth mentioning that medical experts’ marked GT tumors would be the best results that any automatic segmentation could generate, and hence, Table 3 equivalently is to have compared ours with the network trained by using the best possible segmented tumor areas.

**Table 4:** Averaging tumor dice score calculated between medical experts’ marked GT tumor areas and ellipse tumor bounding box areas

Case Study	Dataset	Av. Dice score (∣*σ*∣)
A	US	0.8046 (0.0652)
B	TCGA	0.8279 (0.0514)

### Discussion

Some insights can be obtained from our experimental results using the proposed strategy:

- Using ellipse bounding box strategy showed good performance on two different datasets for diffuse glioma-subtype prediction: US dataset for WHO grade 2 dLGGs (to predict 1p/19q codeletion/non-codeletion) and TCGA data (to predict IDH mutation/wild-type). It is worth mentioning that other shape of bounding boxes, e.g., rectangles [42], can also be selected. We chose elliptical shape to reduce the false positive tumor pixels around the corner areas of rectangles, so that fewer non-tumor pixels would be wrongly labeled and subsequently used for supervised training of tumors.
- The average test accuracy of US dataset is lower comparatively because it consists of only dLGG without significant contrast enhancement. The other reason is rather smaller dataset size. On the other hand, TCGA dataset performs better probably because it consists of patients with both LGG and HGG groups and since the task of IDH detection is easier than that of 1p/19q codeletion.
- Average test accuracy on both the datasets, showed slight degradation in performance of about 3% on the ROIs selected by the proposed strategy. This degradation appears as a trade-off between time and personnel demanding task of manual annotation and a slightly reduced performance and can perhaps be counteracted by having more training data available using this approach.
- Several studies have reported their classification performance on 1p/19q codeletion status using data from both diffuse LGGs and HGGs [10, 12]. Tumors of higher grades typically looks very different and the data is interrelated to molecular markers that it might cause a significant boost in performance. In our study, US dataset consisted of only dLGGs (WHO grade 2) that appears with non-enhanced hyperintensive tumor areas making it more challenging to categorize.
- For IDH genotype, there are some recent studies with superior performance based upon segmentation with more patients and having better balance between classes [13, 43]. Although the scope of the paper was not to compare with them or to create a state-of -the-art prediction. Still, our aim was to study whether a simpler set-up would produce comparable results using a relevant method [44, 45]. Based upon our findings, we believe it is reasonable to use the strategy of tight bounding box and to increase the amount of data available in addition to make it simple and clinical relevant. A significant increase in training data may also actually improve performance in future experiments.

**Limitations:** One effective way of further improving the performance is to increase the size of training dataset since accurate feature characterization in DL is dependent on using large number of training data. In our study, the overall size and the imbalance in the datasets for two classes caused one class with relatively lower performance that has affected the average test performances. One solution is to add synthetic MRI slices in the training dataset through, e.g., employing Generative Adversarial Networks. Furthermore, automatic algorithms instead of manual selection of ellipse bounding box can improve the practical application and could be further studied.

## Conclusion

Manual annotation of MRI tumor areas is time consuming and requires considerable medical expertise. Also, automatic segmentation is ill-defined due to MR image differences from multiple imaging centers. More data is desirable in radiogenomic analysis but many available datasets lack expert tumor boundary annotation. An alternate paradigm of using tumor ROIs by tight ellipse bounding boxes is studied. Our study has shown that it is feasible to use ellipse shape tumor bounding box areas in place of annotated tumor GT areas for supervised trained DL, leading to good performance (average test accuracy of 69.86% for predicting 1p/19q codeletion and 79.50% for IDH mutation) with a small performance degradation (approximately 3.0%). Our results show a possible way to trade-off between training DL schemes using manually annotated tumors and using bounding boxes surrounding the tumors, in terms of saving annotation time and accepting a small performance degradation (about 3%). Our results demonstrate that the tissues surrounding the tumor regions in the ellipse bounding box areas do not cause a major deterioration of performance in predicting the glioma-subtypes.

## Data Availability

Datasets used in the paper was downloaded from TCGA-GBM Collection 10.7937/K9/TCIA.2017.KLXWJJ1Q and TCGA-LGG Collection 10.7937/K9/TCIA.2017.GJQ7R0EF.

## Abbreviations

MRI: Magnetic Resonance Image
dLGG: Diffuse low grade glioma
GT: Ground Truth
DL: Deep Learning
ROIs: Regions of Interest
IDH: Isocitrate dehydrogenase
WHO: World Health Organization
GBM: Glioblastoma
CNN: Convolutional Neural Network
FLAIR: Fluid-Attenuated Inversion Recovery
T1ce: T1 weighted MRI with contrast enhanced
